# *Sargassum fulvellum* Protects HaCaT Cells and BALB/c Mice from UVB-Induced Proinflammatory Responses

**DOI:** 10.1155/2013/747846

**Published:** 2013-07-09

**Authors:** Chan Lee, Gyu Hwan Park, Eun Mi Ahn, Chan-Ik Park, Jung-Hee Jang

**Affiliations:** ^1^Department of Pharmacology, School of Medicine, Keimyung University, 2800 Dalgubeoldaero, Dalseo-gu, Daegu 704-701, Republic of Korea; ^2^Research Institute of Pharmaceutical Sciences, College of Pharmacy, Kyungpook National University, Daegu 702-701, Republic of Korea; ^3^Department of Herbal Foodceutical Science, Daegu Haany University, Gyeongsangbuk-do 712-230, Republic of Korea; ^4^Department of Cosmeceutical Science, Daegu Haany University, 290 Yugok-dong, Gyeongsan-si, Gyeongsangbuk-do 712-230, Republic of Korea

## Abstract

Ultraviolet (UV) radiation has been reported to induce cutaneous inflammation such as erythema and edema via induction of proinflammatory enzymes and mediators. *Sargassum fulvellum* is a brown alga of Sargassaceae family which has been demonstrated to exhibit antipyretic, analgesic, antiedema, antioxidant, antitumor, fibrinolytic, and hepatoprotective activities. The purpose of this study is to investigate anti-inflammatory effects of ethylacetate fraction of ethanol extract of *Sargassum fulvellum* (SFE-EtOAc) in HaCaT keratinocytes and BALB/c mice. In HaCaT cells, SFE-EtOAc effectively inhibited UVB-induced cytotoxicity (60 mJ/cm^2^) and the expression of proinflammatory proteins such as cyclooxygenase-2 (COX-2), tumor necrosis factor-*α* (TNF-*α*), and inducible nitric oxide synthase (iNOS). Furthermore, SFE-EtOAc significantly reduced UVB-induced production of proinflammatory mediators including prostaglandin E_2_ (PGE_2_) and nitric oxide (NO). In BALB/c mice, topical application of SFE-EtOAc prior to UVB irradiation (200 mJ/cm^2^) effectively suppressed the UVB-induced protein expression of COX-2, iNOS, and TNF-*α* and subsequently attenuated generation of PGE_2_ and NO as well. In another experiment, SFE-EtOAc pretreatment suppressed UVB-induced reactive oxygen species production and exhibited an antioxidant potential by upregulation of antioxidant enzymes such as catalase and Cu/Zn-superoxide dismutase in HaCaT cells. These results suggest that SFE-EtOAc could be an effective anti-inflammatory agent protecting against UVB irradiation-induced skin damages.

## 1. Introduction

Skin is the body's largest organ acting as an effective barrier thereby always at a risk of continuous exposure to diverse environmental stimuli including solar ultraviolet (UV). UV radiation is divided into three categories depending on main wavelength ranges such as UVA (long wave, 320–400 nm), UVB (mid wave, 290–320 nm), and UVC (short wave, 200–290 nm) [1]. Particularly, UVB irradiation serves as an important etiologic factor causing inflammatory skin damages, oxidative stress, DNA damage, cellular as well as tissue injuries, cell death, skin cancer, and premature skin aging [1, 2].

UVB exposure on the skin stimulates the inflammatory responses via upregulation of proinflammatory cytokines such as tumor necrosis factor-*α* (TNF-*α*), interleukin-1*α* (IL-1*α*), IL-1*β*, IL-6, and IL-8 [3] and prostaglandins (PGs) such as PGE_2_ and PGD_2_, which were produced by enhanced expression of cyclooxygenase-2 (COX-2) [4]. COX-2 is a rate-limiting enzyme in the biosynthesis of PGs from arachidonic acids and can be stimulated by some cytokines under inflammatory conditions [5]. UVB irradiation also triggers the production of nitric oxide (NO) which is implicated in the pathogenesis of various inflammatory skin disorders [6]. NO is generated by the enzymatic action of inducible nitric oxide synthase (iNOS) which is strongly induced by microbial infection, inflammatory cytokines, and environmental insults in diverse types of cells [6, 7].

Most of the harmful effects of UVB radiation are also associated with the intracellular accumulation of reactive oxygen species (ROS) such as hydrogen peroxide, superoxide anions, and hydroxyl radicals which ultimately lead to oxidative damages [8]. To protect against UVB-induced oxidative stress there are diverse endogenous antioxidant defense systems in our body. However, depletion of endogenous antioxidant enzymes as well as antioxidants in the skin during UVB-mediated oxidative stress results in cellular as well as tissue damages and eventually leads to apoptotic cell death [9].

Therefore, there has been considerable interest in searching for naturally occurring plant products for the prevention and protection from UV-induced skin photodamages. The photoprotective effects of green tea polyphenols, grape seed proanthocyanidins, silymarin, and genistein against UVB-induced skin inflammation, oxidative stress, and DNA damages have been largely studied, and their underlying mechanisms have been suggested using *in vitro* cell cultures as well as *in vivo* animal models [1].

Recently, attention has been focused on extracts, fractions, and single compounds derived from marine algae with antioxidant and anti-inflammatory properties. *Sargassum fulvellum *(SF), a brown alga of Sargassaceae family, is one of the candidates. It has been widely used as a food additive and medicinal purpose in oriental medicine to treat lump, dropsy, swollen, and painful scrotum, and urination problems [10]. Particularly, the dichloromethane extract of SF was reported to have antiedema, antipyretic, and analgesic activities [10]. The freeze-dried SF protected against D-galactosamine-induced hepatopathy in Wistar rats [11]. Polysaccharide fraction obtained by fractional precipitation with ethanol from hot water extract of SF exhibited antitumor activity [12]. In addition, two fibrinolytic compounds were isolated, purified, and characterized from SF [13].

However, the effect of SF on UVB-induced inflammatory skin damages and its underlying molecular mechanisms have not been studied and remain largely unresolved. Therefore, the purpose of this study is to investigate the protective effect of SF against the UVB-induced pro-inflammatory responses *in vitro* HaCaT human keratinocytes as well as *in vivo* BALB/c mice and to further elucidate its antioxidant potentials as a possible mechanism of skin protection.

## 2. Materials and Methods

### 2.1. Plant Materials

Thalli of *Sargassum fulvellum* (SF) was purchased from JukDo market (Pohang, Korea), and voucher specimen is deposited in Department of Cosmeceutical Science, Daegu Haany University (Gyeongsan, Korea). Dried SF (150.0 kg) powder was extracted with 95% ethanol (EtOH) for 72 h at room temperature and then evaporated *in vacuo*. The final yield was 0.29%. The ethanol extract of SF was further successively partitioned with water (300 mL), ethylacetate (EtOAc, 300 mL × 3), and *n*-butanol (*n*-BuOH, 300 mL × 3). The ethylacetate fraction of SF ethanol extract (SFE-EtOAc) was dried in a rotary evaporator. SFE stock solution was prepared with dimethyl sulfoxide (DMSO) and acetone-olive oil (3 : 1) for the cell cultures and animal experiments, respectively.

### 2.2. Chemical Materials

Cell culture medium (Dulbecco's modified Eagle's medium, DMEM), fetal bovine serum (FBS), and antibiotics (penicillins/streptomycin) were purchased from Gibco BRL (Rockville, MD, USA). Primary anti-COX-2, anti-TNF-*α*, and anti-iNOS antibodies and horseradish peroxidase-conjugated secondary anti-rabbit antibody were the products of Santa Cruz Biotechnology (Santa Cruz, CA, USA). Antiactin antibody, MTT [3-(4,5-dimethylthiazol-2-yl)-2,5-diphenyl tetrazolium bromide], and other chemicals were obtained from Sigma-Aldrich (St. Louis, MO, USA). 2′7′-Dichlorofluroscein diacetate (DCF-DA) dye was supplied from Invitrogen Co. (Carlsbad, CA, USA).

### 2.3. Cell Culture and Treatment

Immortalized human keratinocytes, HaCaT cells were cultured in DMEM supplemented with 10% fetal bovine serum and antibiotic mixture (100 units/mL penicillin and 100 *μ*g/mL streptomycin) and routinely maintained in a humidified atmosphere containing 5% CO_2_-95% air at 37°C. The culture medium was changed every other day, and cells were seeded at the appropriate density for further experiments. HaCaT cells were pretreated with ethylacetate fraction of SF ethanol extract (SFE-EtOAc) for 9 h and then exposed to UVB (280–320 nm, peak emission at 312 nm, 60 mJ/cm^2^) using a UVB irradiation equipment (BLX-E254, Vilber Lourmat, France). During the UVB exposure, cells were kept in a thin layer of phosphate-buffered saline (PBS), and then, PBS was replaced with culture medium containing 1% DMSO (vehicle control) or SFE-EtOAc.

### 2.4. Experimental Animals and Treatment

BALB/c mice (male, 6–8 weeks old) were purchased from DaeHan Biolink Co., Ltd. (Daegu, Korea). The animals were maintained in standard conditions (21–25°C temperature, 40–60% humidity, and 12 h light-12 h dark cycle) with free access to foods and water. Animals were housed and handled in accordance with the Institutional Animal Care and Use Guidelines of the Daegu Haany University and National Institute of Health. After shaving the dorsal side of the skin by a clipper, mice in the resting phase of the hair cycle were used in the following experiments. Vehicle (acetone-olive oil, 3 : 1) or SFE-EtOAc was topically applied to the back of each mouse, and after 30 min, mice were exposed to 200 mJ/cm^2^ UVB (BLX-E254) in a specially designed cage. The exact dosage of UVB was closely monitored by spectroradiometer (UVP, CA, USA).

### 2.5. Cell Viability Assay (MTT Dye Reduction Assay)

To determine the safety and protective effect of SFE-EtOAc, cell viability was measured by MTT assay which is based on the mitochondrial enzyme-dependent reduction of MTT dye to purple formzan. After incubation of UVB-exposed HaCaT cells with or without SFE-EtOAc, MTT solution (5 mg/mL in PBS) was added to each well and additionally treated for 2 h. Finally, the medium was discarded and the formazan products were dissolved by DMSO. The optical density (OD) at 540 nm was measured by a microplate reader (Molecular Device, CA, USA). Cell viability was represented as % by calculating the relative OD ratio of each experimental group to the corresponding control. The mean OD of the control group was assigned as 100%.

### 2.6. Western Blot Analysis

Harvested cells and pulverized dorsal skin samples were lysed and homogenated with RIPA buffer (150 mM NaCl, 0.5% Triton X-100, 50 mM Tris-HCl (pH 7.4), 20 mM EGTA, 1 mM dithiothreitol, and 1 mM Na_3_VO_4_) containing protease inhibitor cocktail (Roche Diagnostics, Mannheim, Germany). Protein samples were prepared by centrifugation at 14,000 g for 15 min, and their concentrations were determined by BCA protein assay (Pierce, Rockford, IL, USA). The proteins were electrophoresised on SDS-PAGE gels and blotted onto polyvinylidene fluoride (PVDF) membranes. The membranes were blocked with 5% skim milk in PBS containing 0.01% Tween 20 (PBST) and subsequently incubated with primary antibodies overnight at 4°C. The immunoreactive bands were detected by using horseradish peroxidase- (HRP-) conjugated secondary antibody and enhanced chemiluminescence (ECL) reagent (Amersham Bioscience, NJ, USA). The images were captured and analyzed by ImageQuant LAS 4000 Multi Gauge software (Fujifilm, Tokyo, Japan). Data are representative of three independent experiments, which gave rise to a similar trend. 

### 2.7. PGE_2_ Assay

 The PGE_2_ levels in the culture medium and serum were determined by a commercially available PGE_2_ enzyme immunoassay (EIA) kit (Cayman Chemical, CA, USA) according to the manufacturer's instructions. In brief, after treatment of SFE-EtOAc and irradiation with UVB, the culture medium and serum were collected and transferred to a plate precoated with goat polyclonal anti-mouse IgG. In the next step, anti-PGE_2_ monoclonal antibody and acetylcholinesterase linked to PGE_2_ (AchE tracer) were added and incubated for 18 h at 4°C. The plate was rinsed three times with washing buffer and then reacted with Ellman's reagent for 1 h at room temperature. The absorbance of mixture at 405 nm was monitored by spectrophotometric microplate reader. The relative PGE_2_ concentrations were calculated by using PGE_2_ as a standard.

### 2.8. Nitrite Assay

As NO in itself is labile with very short half life, to determine NO generation, the concentrations of nitrite in the medium and serum were measured by Griess assay. The collected culture medium and serum were mixed with the same volume of Griess reagent (0.1% *N*-(1-naphthyl) ethylendiamine and 1% sulfanilamide in 5% phosphoric acid) and incubated for 10 min at room temperature. The absorbance of mixture at 540 nm was determined by microplate reader. The relative nitrite concentrations were calculated by using sodium nitrite (NaNO_2_) as a standard.

### 2.9. Quantification of Intracellular Reactive Oxygen Species (ROS) Levels

The intracellular accumulation of ROS was measured by DCF-DA dye. DCF-DA easily diffuses into the cell and then is hydrolyzed by an intracellular esterase to DCF which can react with peroxides to produce fluorescence. SFE-EtOAc or vehicle-pretreated and UVB-exposed HaCaT cells were loaded with DCF-DA solution (50 *μ*M in PBS) for 15 min, washed three times in PBS, and lysed with DMSO. The fluorescence intensity was monitored by fluorescence microplate reader (Gemini XS, Molecular Devices Co., USA) with excitation at 488 nm and emission at 535 nm. The fluorescence values were expressed as a percentage considering nonirradiated DCF-DA-stained cells as 100%.

### 2.10. Reverse-Transcriptase Polymerase Chain Reaction (RT-PCR)

The mRNA expression of antioxidant enzymes was assessed by RT-PCR. Total RNA was extracted using Trizol reagent (Invitrogen Co., CA, USA) and then converted to cDNA using a Reverse Transcription System (Promega, Madison, WI, USA). The target cDNA was amplified by using the following primers: catalase (CAT), forward 5′-CCG ACG AGA TGG CAC ACT TTG ACA-3′ and reverse 5′-CGC GAG CAC GGT AGG GAC AGT TC-3′; copper/zinc-superoxide dismutase (Cu/Zn-SOD), forward 5′-CCA TCA ATA TGG GGA CAA TAC AC-3′ and reverse 5′-ACA CGA TCT TCA ATG GAC AC-3′; glyceraldehyde 3-phosphate dehydrogenase (GAPDH), forward 5′-GCC AAG GTC ATC CAT GAC AAC-3′ and reverse 5′-AGT GTA GCC CAG GAT GCC CTT-3′. The amplification was carried out at 95°C for 30 s (denaturation), 61°C (CAT), 49°C (Cu/Zn-SOD), 57°C (GAPDH) for 1 min (annealing), and 72°C for 1 min (extension). After 33 cycles of reactions, the PCR products were separated by electrophoresis on 1.5% agarose gel at 50 V for 30 min. Gels were then stained with ethidium bromide (EtBr) and visualized by UV light using Quantity One Software of Image analysis Gel Doc XR System (BIO-RAD, CA, USA).

### 2.11. Statistical Analysis

Each data point represents the mean ± SD from at least three independent experiments. Statistical comparisons among groups were conducted by one-way analysis of variance (ANOVA) followed by Tukey's *post hoc* test. A value of *P* less than 0.05 was regarded as statistically significant in all experiments.

## 3. Results

### 3.1. Attenuation of UVB-Induced Cytotoxicity by Ethylacetate Fraction of SF Ethanol Extract in HaCaT Cells

**Figure 1:**
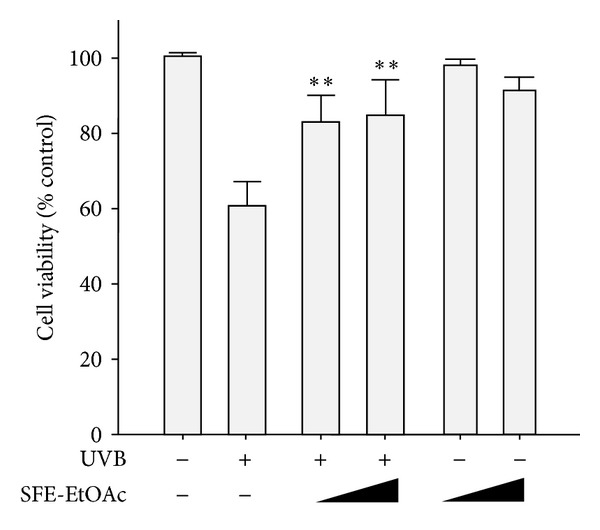
Protective effect of ethylacetate fraction of SF ethanol extract (SFE-EtOAc) against UVB-induced cytotoxicity in HaCaT cells. HaCaT cells were preincubated with vehicle or SFE-EtOAc (30 *μ*g/mL and 100 *μ*g/mL) for 9 h prior to UVB (60 mJ/cm^2^) exposure. Twenty-four hours after UVB exposure, cell viability was measured by the MTT dye reduction assay. Cell viability is expressed as the percentage of control. Data are shown as mean ± SD values (*n* = 3). **P < 0.01 compared with UVB-exposed group.

To assess the protective effect of ethylacetate fraction of SF ethanol extract (SFE-EtOAc) on the UVB-induced cytotoxicity, relative cell viability was determined by MTT dye reduction assay. The UVB irradiation (60 mJ/cm^2^) alone decreased the cell viability in HaCaT human keratinocytes when compared with the vehicle-treated control group (1% DMSO), which was significantly restored by pretreatment with SFE-EtOAc in a concentration-dependent manner (30 *μ*g/mL and 100 *μ*g/mL) (Figure 1). Under the same experimental condition, we confirmed that vehicle alone did not cause any significant alterations in the cell viability. SFE-EtOAc substantially ameliorated UVB-induced cytotoxicity without exhibiting apparent self-toxicity when treated alone (Figure 1). This result provides cytoprotective potential of SFE-EtOAc against UVB irradiation-induced cellular damage and death.

### 3.2. Inhibitory Effect of SFE-EtOAc on UVB-Induced Expression of COX-2 and TNF-*α* and Production of PGE_2_ in HaCaT Cells

**Figure 2:**
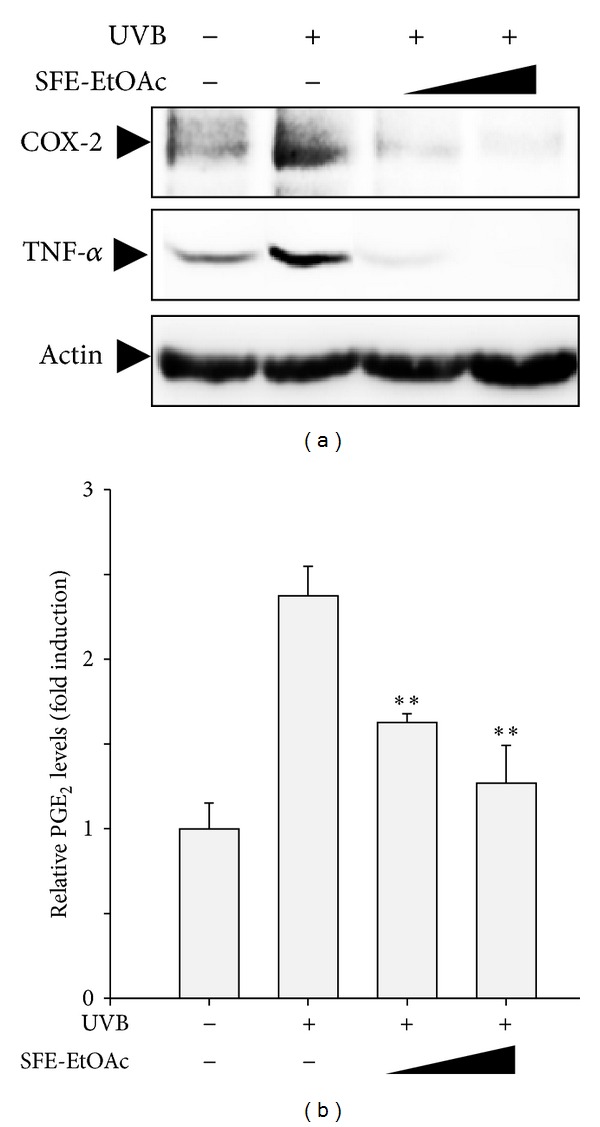
Inhibition of UVB-induced COX-2 and TNF-*α*expressions and subsequent PGE_2_ production by SFE-EtOAc in HaCaT cells. HaCaT cells were pretreated with vehicle or SFE-EtOAc (30 *μ*g/mL and 100 *μ*g/mL) for 9 h before irradiation of UVB (60 mJ/cm^2^). Twenty-four hours after UVB exposure (60 mJ/cm^2^), the protein expression of COX-2 and TNF-*α* (a) and accumulation of PGE_2_ into the media (b) were monitored by western blot analysis and ELISA, respectively. (a) Protein extracts were loaded onto a SDS-PAGE and then transferred to PVDF membranes. The membranes were probed with anti-COX-2, anti-TNF-*α*, or antiactin primary antibody. Actin levels were assessed to confirm the equal amount of protein loading. Representative blots from three independent experiments are shown. (b) The relative levels of the PGE_2_ were represented as fold induction in comparison with the vehicle-treated control cells. Asterisks indicate a significant difference from the group exposed to UVB alone (**P < 0.01).

To analyze whether SFE-EtOAc could inhibit UVB-induced pro-inflammatory damages in HaCaT cells, the protein expression of COX-2 and TNF-*α* and subsequent production of PGE_2_ were measured by western blot analysis and ELISA, respectively. The maximal induction time point for the protein expression of COX-2 and TNF-*α* by UVB irradiation (60 mJ/cm^2^) was determined by kinetic profiles of western blot analysis. Single UVB exposure markedly enhanced protein levels of COX-2 and TNF-*α* (Figure 2(a)) and increased PGE_2_ levels released into the media (Figure 2(b)) at 24 h, which were effectively suppressed by increasing concentrations of SFE-EtOAc pretreatment (30 *μ*g/mL and 100 *μ*g/mL). At 100 *μ*g/mL of SFE-EtOAc, the COX-2 and TNF-*α* expression (Figure 2(a)) and PGE_2_ production (Figure 2(b)) were almost abolished to vehicle-treated control levels. These results suggest a strong anti-inflammatory potential of SFE-EtOAc against UVB irradiation.

### 3.3. Inhibitory Effect of SFE-EtOAc on UVB-Induced iNOS Expression and NO Production in HaCaT Cells

**Figure 3:**
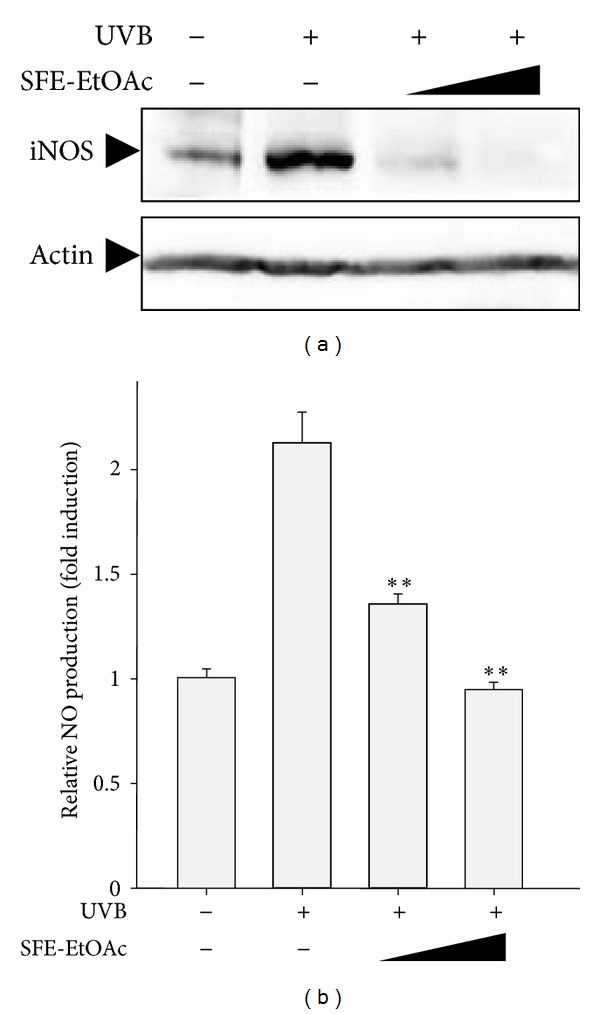
Inhibition of UVB-induced iNOS expression and subsequent NO production by SFE-EtOAc in HaCaT cells. HaCaT cells were pretreated with vehicle or SFE-EtOAc (30 *μ*g/mL and 100 *μ*g/mL) for 9 h and then exposed to UVB (60 mJ/cm^2^). Thereafter, media were replaced with fresh media containing SFE-EtOAc or vehicle, and cells were additionally incubated for 24 h. (a) Western blot analysis was performed using anti-iNOS or antiactin primary antibody. Representative blots from three independent experiments are shown. The actin levels were monitored for the confirmation of equal amount of protein loading. (b) The NO levels released into the medium were measured by Griess assay as described in Section 2. The relative levels of the NO production were represented as fold induction in comparison with the vehicle-treated control cells (4.28 ± 0.18 *μ*M). Asterisks indicate a significant difference compared with UVB-irradiated group (**P < 0.01).

To further examine the anti-inflammatory activity of SFE-EtOAc against UVB irradiation, the protein levels of iNOS and subsequent generation of NO were assessed by western blot analysis and Griess assay, respectively. The optimal time to compare the protein expression of iNOS was determined based on a time course profile of western blot analysis. When HaCaT keratinocytes were exposed to UVB (60 mJ/cm^2^), iNOS protein expression was markedly increased and SFE-EtOAc pretreatment (30 *μ*g/mL and 100 *μ*g/mL) before UVB irradiation resulted in an effective inhibition of iNOS protein levels (Figure 3(a)). Furthermore, the amount of NO released into the media was relatively higher in the UVB-irradiated group, which was significantly reduced by pretreatment with SFE-EtOAc (Figure 3(b)).

### 3.4. Inhibitory Effect of SFE-EtOAc on UVB-Induced Expression of COX-2 and TNF-*α* and Production of PGE_2_ in BALB/c Mice

**Figure 4:**
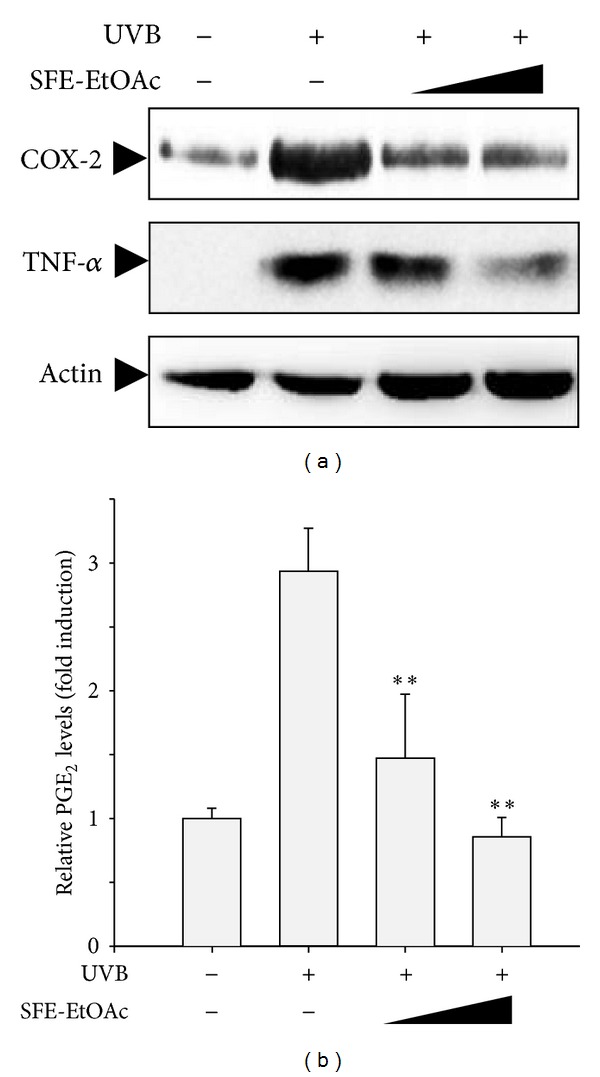
Inhibition of UVB-induced COX-2 and TNF-*α* expression and subsequent PGE_2_ production by SFE-EtOAc in mouse skin. SFE-EtOAc (3 *μ*g and 10 *μ*g) dissolved in acetone-olive oil (1 : 3) was topically applied to BALB/c mice 30 min before UVB (200 mJ/cm^2^) stimulation. Twenty-four hours later, skin samples were excised from the backs of mice. (a) Protein levels of COX-2 and TNF-*α* were determined by western blot analysis using anti-COX-2 and anti-TNF-*α* antibodies. Actin was detected to verify equal protein loading. (b) The PGE_2_ levels in serum were measured by ELISA as described in Section 2. The relative PGE_2_ levels were shown as fold induction from the vehicle-treated sham control. Asterisks indicate a significant difference from the value obtained with UVB alone (**P < 0.01). For sham control and UVB alone-irradiated groups, same amount of vehicle (acetone-loive oil) was topically applied instead of SFE-EtOAc solution.

In order to confirm the anti-inflammatory effect of SFE-EtOAc against UVB-stimulated inflammatory responses *in vivo*, BALB/c mice were exposed to UVB (200 mJ/cm^2^) with or without pretreatment of SFE-EtOAc (3 *μ*g and 10 *μ*g) for 30 min. After UVB irradiation the protein expression of COX-2 and TNF-*α* in the mouse skin was markedly increased when compared with vehicle-treated control animals (acetone-olive oil 3 : 1) (Figure 4(a)). The elevated protein levels of COX-2 and TNF-*α* were gradually decreased by topical application of SFE-EtOAc in a dose-related manner (Figure 4(a)). In accordance with COX-2 protein expression, SFE-EtOAc treatment significantly downregulated UVB-induced PGE_2_ production in BALB/c mice almost to control levels (Figure 4(b)).

### 3.5. Inhibitory Effect of SFE-EtOAc on UVB-Induced iNOS Expression and NO Production in BALB/c Mice

**Figure 5:**
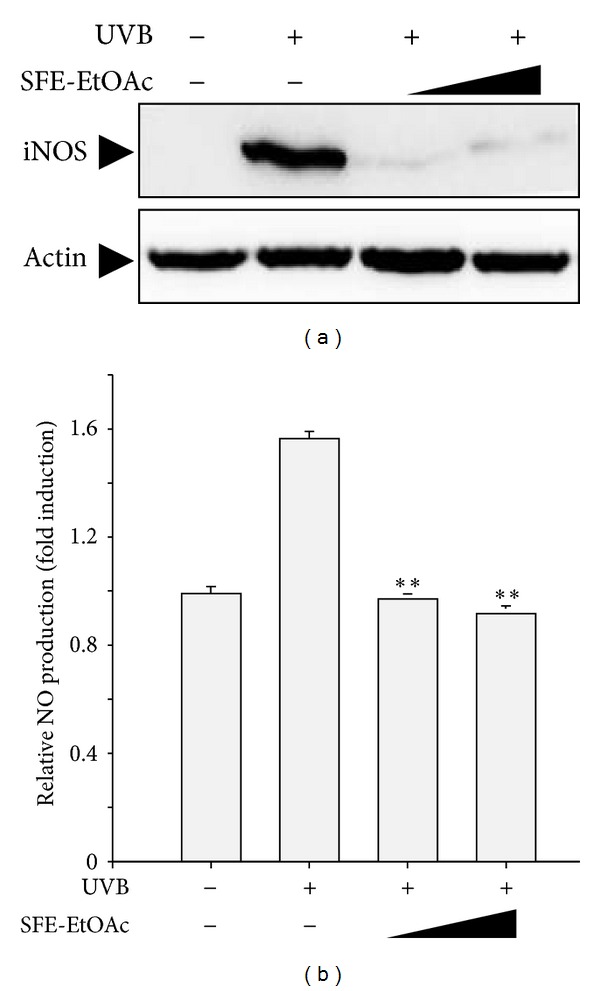
Inhibition of UVB-induced iNOS expression and subsequent NO production by SFE-EtOAc in mouse skin. Prior to UVB (200 mJ/cm^2^) irradiation SFE-EtOAc (3 *μ*g and 10 *μ*g) was topically applied on the back of BALB/c mice for 30 min. Twenty-four hours later, skin samples were excised from the backs of mice. (a) Protein expression of iNOS was determined by western blot analysis using iNOS-specific primary antibody. Actin was detected to verify equal protein loading. (b) The NO levels in serum were assessed by Griess assay as indicated in Section 2. The relative NO production was represented as fold induction in comparison with the vehicle-treated sham control group. Asterisks indicate a significant difference from the value obtained with UVB alone (**P < 0.01). For sham control and UVB alone-irradiated groups, same amount of vehicle (acetone-loive oil) was topically applied instead of SFE-EtOAc solution.

To investigate the inhibitory effect of SFE on the UVB-induced iNOS expression and NO production, BALB/c mice were pretreated with vehicle or SFE-EtOAc (3 *μ*g and 10 *μ*g) for 30 min before exposure to UVB (200 mJ/cm^2^). UVB irradiation alone increased protein expression of iNOS comparing with vehicle-treated control animals (acetone-olive oil 3 : 1), whereas topical application of SFE-EtOAc effectively suppressed UVB-elevated protein levels of iNOS as assessed by western blot analysis (Figure 5(a)). Alike result of iNOS expression, SFE-EtOAc significantly inhibited the production of NO to a level commensurate with UVB-unstimulated vehicle-treated control (Figure 5(b)).

### 3.6. Augmentation of Cellular Antioxidant Defense Enzymes by SFE-EtOAc

**Figure 6:**
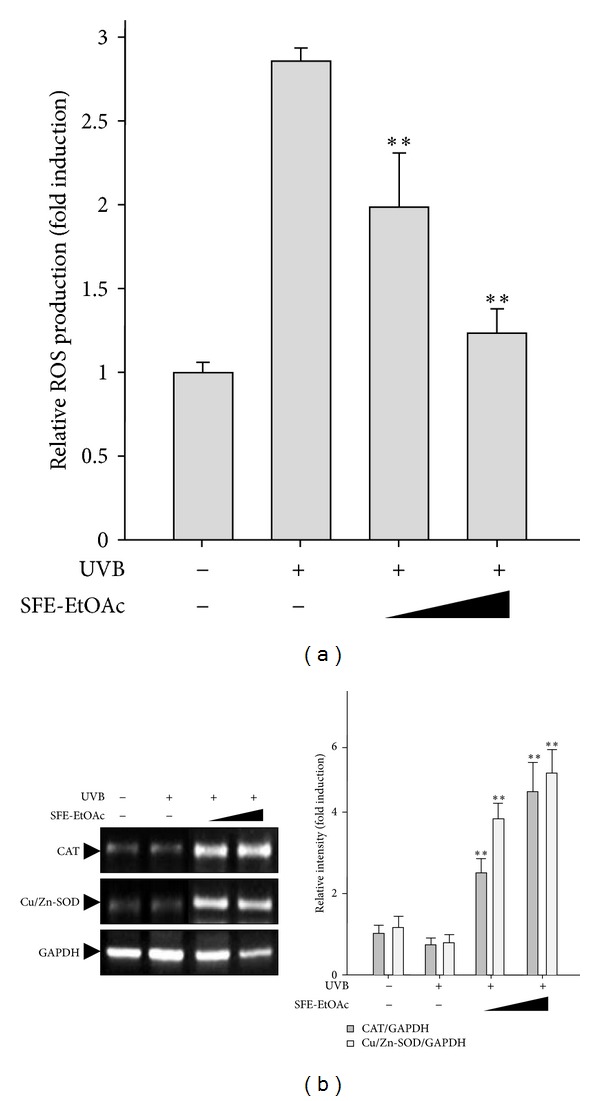
Effect of SFE-EtOAc on the intracellular ROS levels and mRNA expression of antioxidant enzymes in HaCaT cells. HaCaT cells were pretreated with vehicle or SFE-EtOAc (30 *μ*g/mL and 100 *μ*g/mL) for 9 h and then stimulated with UVB (60 mJ/cm^2^). (a) Intracellular ROS generation was measured by relative fluorescence intensity using DCF-DA dye as indicated in Section 2. Data are shown as mean ± SD values (*n* = 3). **P < 0.01 compared with UVB-exposed group. (b) The mRNA levels of antioxidant enzymes such as catalase and Cu/Zn-SOD were measured by RT-PCR using their specific primers. Quantification data of mRNA expression of antioxidant enzymes analyzed by RT-PCR, which was normalized by *GAPDH* levels as loading control. Asterisks indicate a significant difference compared with UVB-irradiated group (**P < 0.01).

When the skin is exposed to excess UVB, as one of the first events, ROS is generated in keratinocytes, which consequently leads to pro-inflammatory process and other biochemical reactions related to oxidative stress. Therefore, in the next experiment, to evaluate the antioxidant activity of SFE-EtOAc as a protective molecular mechanism, intracellular accumulation of RSO and the mRNA expression of antioxidant enzymes were measured by conducting DCF-DA staining and RT-PCR, respectively. In HaCaT cells, SFE-EtOAc (30 *μ*g/mL and 100 *μ*g/mL) pretreatment effectively reduced UVB-increased intracellular ROS levels (Figure 6(a)) and highly upregulated mRNA levels of antioxidant enzymes such as CAT and Cu/Zn-SOD (Figure 6(b)). These results suggest that SFE-EtOAc exhibits strong antioxidant potential via fortifying cellular antioxidant defense capacity thereby possibly protecting against UVB-induced inflammatory and oxidative damages.

## 4. Discussion

Exposure to solar UVB radiation induces an array of adverse reactions in the skin such as edema, erythema, hyperplastic responses, sunburn, photoaging, and cancer via altered intracellular signaling related with inflammation and oxidative stress. Therefore, the amelioration of UVB-induced inflammatory and oxidative responses represents a potential strategy for the prevention and protection against UVB-induced cellular and tissue photodamages. In this regards, recently a wide variety of edible and medicinal plants have been investigated for topical application. The present study demonstrates the inhibitory effect of *Sargassum fulvellum* (SF) against UVB-induced proinflammatory skin damages, suggesting the potential photoprotective effect of SF in UVB-irradiated HaCaT cells *in vitro* as well as mouse skin *in vivo*. 

Keratinocytes respond to the major changes in the inflammation and immunomodulation observed after UVB exposure, at least in part via the UVB-induced expression of inflammatory enzymes and release of pro-inflammatory mediators. COX-2 plays a key role in acute UVB-induced inflammation by catalyzing the generation of PGE_2_ from prostanoid precursors. The present study demonstrates that in human keratinocyte HaCaT cells and BALB/c mouse skin, ethylacetate fraction of SF ethanol extract (SFE-EtOAc) effectively inhibited UVB-triggered COX-2 expression and subsequent PGE_2_ synthesis. 

In accordance with our experimental data, the expression of COX-2 is significantly elevated in HaCaT human keratinocytes [14–16] and JB6 P+ mouse epidermal cells [17] after a single exposure to UVB and sulforaphane [14], *γ*-tocotrienol [15], luteolin [16], and delphinidin [17] have been shown to suppress UVB-induced COX-2 expression and subsequent formation of PG metabolites. Stimulation of HR-1 [14, 15, 18, 19] and SKH-1 [20] hairless mouse skin with UVB resulted in a significant increase in COX-2 expression and conversely topical application of sulforaphane [14], *γ*-tocotrienol [15], anthocyanins from black soybean seed coats [18], oligonol [19], and proanthocyanidins from grape [20] inhibited UVB-induced COX-2 expression.

Furthermore, exposure of the skin to UVB radiation is known to enhance the levels of pro-inflammatory cytokines. As keratinocytes are considered to be major sources of cytokines, chemokines, growth factors, and many others, UVB radiation can cause a series of changes in the cutaneous cytokine micromilieu such as TNF-*α*, IL-1 *α*, IL-1*β*, IL-6, and IL-8 [3]. In this study, UVB markedly induced the protein expression of TNF-*α* in HaCaT cells and BALC/c mice, which was effectively suppressed by pretreatment of SFE-EtOAc. TNF-*α* is a major cytokine involved in the early stage of inflammation released when keratinocytes are damaged and stimulates neighboring keratinocytes to amplify the responses.

In line with our notion, previous *in vitro* and *in vivo* laboratory studies have shown the photoprotective effects of natural products by suppressing UVB-induced expression of diverse cytokines. Water extract of *Zingiber officinale*, gingerol, and shogaol inhibited production of cytokines such as TNF-*α*, IL-1*β*, IL-6, and IL-8 in UVB-irradiated HaCaT cells and hyperplasia, infiltration of leukocytes, and dilation of blood vessels in UVB-exposed C57BL/6 mice [3]. Tannic acids attenuated UVB-induced cutaneous inflammation in HaCaT cells by inhibiting UVB-enhanced expression of cytokines including TNF-*α*, IL-1*β*, IL-6, and IL-18 [21].

In addition, iNOS expression and activity have been reported to play an important role in various skin disorders such as sunburn erythema, psoriasis, cutaneous lupus erythematosus, and squamous cell carcinoma [6, 22]. The expression of iNOS is induced in response to microbial infection, cytokine stimulation, and environmental insults in a variety of cell types including keratinocytes and subsequently involved in the release NO [6]. NO is first recognized as a paracrine regulator of diverse biological responses such as smooth muscle relaxation, vasodilation, neurotransmission, immunomodulation, and modification of cellular proliferation [6]. However, excess production of NO can exert toxic effects via induction of oxidative stress, DNA damage, and apoptosis by its direct action or indirect formation of more potent oxidant, peroxynitrite (ONOO^−^) [23]. 

Our findings demonstrate that UV radiation is capable of induction of iNOS accompanying subsequent NO release in HaCaT keratinocytes and BALB/c mice, and SFE-EtOAc treatment prior to UV exposure markedly decreased UVB-induced expression of iNOS and production of NO. Treatment of human keratinocytes with amniotic membrane extract [7] and human melanocytes with ferulic acid ethyl ester [24] also has been shown to result in a reduction of iNOS expression as well as NO generation thereby protecting cells from UVB-induced oxidative damage and cell death. 

The UVB-induced inappropriate overexpression of COX-2, cytokines, and iNOS results from dysregulation of intracellular signal transduction pathways mediated by various transcription factors and upstream kinases. Previous studies demonstrated that NF-*κ*B activation was observed in UVB-stimulated HaCaT cells *in vitro* and mouse skin *in vivo*. In HaCaT cells, [6]-gingerol, a pungent ingredient of ginger [2], and docosahexaenoic acid, a representative *ϖ*-3 polyunsaturated fatty acid in fish oil [25], effectively reduced UVB-induced expression of COX-2 by inhibiting NF-*κ*B activation via suppression of I*κ*B*α* phosphorylation. Epigallocatechin-3-gallate, a green tea polyphenol, downregulated the UVB-induced iNOS mRNA synthesis and NO generation by decreasing activation and translocation of NF-*κ*B [26]. Moreover, curcumin, a yellow pigment present in the rhizome of turmeric, inhibited UVB-induced expression of cytokines by suppressing the DNA binding of NF-*κ*B in HaCaT cells [27].

UVB irradiation results in local inflammation, which further amplifies ROS generation. ROS has been suggested to play an important role in the UVB-induced activation of COX-2. Pretreatment with HaCaT cells with an antioxidant *N*-acetylcysteine partly inhibited UVB-induced COX-2 expression [28]. Conversely, in human epidermoid keratinocytes, depletion of endogenous antioxidant reduced glutathione (GSH) augmented UVB-upregulated COX-2 expression [29]. To protect against ROS-mediated oxidative stress, the skin possesses an array of antioxidant defense system. However, chronic and excess exposure to UV radiation can overwhelm the antioxidant capacity and lead to oxidative damages to critical cellular components such as mitochondria, DNA, lipid membranes, and proteins [8]. 

In HaCaT cells, SFE-EtOAc enhanced mRNA expression of antioxidant enzymes such as CAT and Cu/Zn-SOD and augmented cellular defense capacity against oxidative stress. Catalase catalyzes the decomposition of hydrogen peroxide (H_2_O_2_) to water and oxygen molecules. SOD catalyzes the dismutation of superoxide anion (O_2_^−·^) to hydrogen peroxide thereby regulating intracellular superoxide anion levels [22]. Cu/Zn-SOD isoform exists at high levels throughout the cells particularly in the cytosol. 

In line with our findings, ethyl acetate fraction of *Sargassum muticum *protected against UVB-induced oxidative damages by restoration of protein expression and activity of antioxidant enzymes such as CAT and Cu/Zn-SOD in HaCaT cells [9]. Moreover, methanol extract of *Sargassum thunbergii* protected against hydrogen peroxide-induced cytotoxicity and oxidative stress by increasing the expression of antioxidative enzymes including SOD, CAT, glutathione peroxidase (GPx), and glutathione reductase (GR) [30]. Previous studies have shown that extracts of three varieties of leaves from *Lepidium meyenii* prevented the UVB-developed sunburn, epidermal hyperplasia, leukocytic infiltration, and other alterations via augmenting antioxidant activity of SOD and CAT in male Swiss mice [31]. In human skin dermal fibroblast adult cells, the activities of enzymatic antioxidants (SOD, CAT, and GPx) and the levels of nonenzymatic antioxidants (GSH) were markedly decreased in UVB-irradiated cells, which were effectively restored by sesamol pretreatment leading to cellular protection against UVB-induced oxidative damages and apoptotic cell death [32]. 

However, to elucidate the intracellular signaling pathways involved in the antioxidant effect of SFE-EtOAc in HaCaT cells and to further confirm its antioxidant molecular mechanisms in BALB/c mice, additional research targeting redox-sensitive transcription factors and upstream regulators should be conducted. Moreover, particularly in animal models, the possibility of SFE-EtOAc acting as a UVB blocker is not ruled out and should be verified in concert with its antioxidant potentials.

## 5. Conclusions

SFE-EtOAc inhibited UVB-induced skin inflammation such as enhanced protein expression of COX-2, TNF-*α*, and iNOS and subsequent production of PGE_2_ and NO *in vitro* HaCaT human keratinocytes as well as *in vivo* BALB/c mice, which would provide a novel insight into the application of SFE-EtOAc for antiphotoinflammatory purposes. The anti-inflammatory effect of SFE-EtOAc seemed to be mediated by, at least in part, attenuation of oxidative stress via upregulation of cellular antioxidant defense enzymes. Therefore, the topical treatment of SFE-EtOAc or routine consumption of SFE-EtOAc containing functional foods in combination with sunscreens or skin care products may be an efficient strategy for mitigating the harmful effects of excessive UVB exposures.
